# Long noncoding RNA HULC regulates the NF‐κB pathway and represents a promising prognostic biomarker in liver cancer

**DOI:** 10.1002/cam4.5263

**Published:** 2022-10-10

**Authors:** Shihai Liu, Lakshmi Huttad, Guifang He, Weitai He, Changchang Liu, Duo Cai, Hao Chen, Jing Qiu

**Affiliations:** ^1^ Medical Animal Lab The Affiliated Hospital of Qingdao University Qingdao China; ^2^ Asian Liver Center, Department of Surgery, School of Medicine Stanford University Stanford California USA; ^3^ National Engineering Laboratory for Resource Development of Endangered Crude Drugs in Northwest of China, College of Life Sciences Shaanxi Normal University Xi'an China; ^4^ Key Laboratory of Biomedical Information Engineering of Ministry of Education, Biomedical Informatics & Genomics Center, School of Life Science and Technology Xi'an Jiaotong University Xi'an China; ^5^ Research Institute of Xi'an Jiaotong University Hangzhou Zhejiang China; ^6^ Department of Stomatology Qingdao Municipal Hospital Qingdao China

**Keywords:** hepatocellular carcinoma, HULC; autophagy, long noncoding RNAs, NF‐kB pathway

## Abstract

**Background:**

Long noncoding RNAs (lncRNAs) are involved in a diverse array of biological processes. While lncRNAs are commonly upregulated in hepatocellular carcinoma (HCC), the specific regulatory roles they play in this oncogenic context require further study and clarification. Although HULC (lncRNA highly upregulated in liver cancer) is involved in disease pathogenesis, its precise role in this context remains unclear.

**Methods:**

Here, we have explored the mechanistic relevance of HULC expression by assessing its expression in patient samples. The importance of this lncRNA in the onset and progression of HCC was investigated through in vitro approaches including Western blotting, quantitative PCR, Transwell assays, electron microscopy, wound healing assays, and real‐time cell analysis (RTCA). Additionally, the in vivo functions of this lncRNA were assessed using an orthotopic HCC xenograft in nude mouse model system.

**Results:**

HULC was identified as a lncRNA that is highly upregulated in human liver tumors. In vitro, HULC was able to promote HCC malignancy, although its excess overexpression also led robust autophagic induction, promoting the increased expression of autophagy‐associated genes including LC3 and Beclin‐1. At a mechanistic level, HULC was able to promote the phosphorylation of p65 and IkBkB thus enhancing autophagy by increasing LC3II levels in a manner dependent upon the NF‐κB pathway. HULC downregulation was also linked to impaired orthotopic HCC tumor growth in vivo*.* The link between HULC and autophagy may play a role in disease progression.

**Conclusions:**

These results suggest that HULC is an oncogenic lncRNA, and may thus offer value as a prognostic biomarker and promoter of HCC development, in addition to being a potential therapeutic target in this cancer type.

## Graphical abstract

HULC, being oncogenic lncRNA, is a prognostic promoter of HCC.

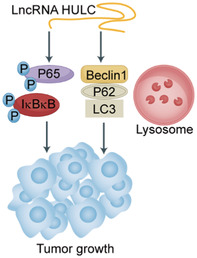

## 1 INTRODUCTION

Hepatocellular carcinoma (HCC) is the third most common cause of cancer‐associated mortality, with a poor prognosis and high relapse rates such that it remains a persistent threat to global public health.^1^, ^2^ Hepatocarcinogenesis is a process that is influenced by a range of genetic and environmental risk factors, such as hepatitis virus infection, alcohol abuse, and aflatoxin ingestion,^3^, ^4^ with tumor development proceeding via a multi‐stage process wherein genetic and epigenetic changes ultimately subvert normal cellular function in favor of malignancy.^5^ At present, surgical tumor resection represents the only reliable approach to achieving prolonged survival for most HCC patients, and roughly two‐thirds of these patients ultimately suffer from tumor recurrence.^6^ A majority of HCC patients are diagnosed with advanced disease, and despite extensive research regarding the mechanistic basis for this deadly cancer type, mortality rates remain high.

Long noncoding RNAs (lncRNAs) can regulate a wide range of biological processes, and play key pathological roles in cancer and other diseases wherein they modulate apoptosis, cellular proliferation, differentiation, and autophagy.^7^, ^8^, ^9^ Highly upregulated in liver cancer (HULC), encoded on chromosome 6p24.3, is a lncRNA that has been reported to be upregulated in liver cancer. HULC transcription produces a ~500 nt RNA that is located in the cytoplasm and participates in the development of HCC.^10^ Matouka et al. observed HULC upregulation in HBV‐producing cell lines,^11^ while Du et al. determined that HBx‐mediated HULC expression drives enhanced liver cell proliferation by inhibiting the tumor suppressor gene p18.^12^ Wang et al. further detected a regulatory interaction between HULC and CAMP‐responsive element‐binding protein (CREB) whereby HULC can sequester and downregulate miR‐372, thus reducing the translational inhibition of the target gene PRKACB, in turn promoting the phosphorylation of CREB.^13^ The silencing of HULC has previously been shown to impair gastric cancer cell growth and associated drug resistance.^14^ HULC can drive the onset of multiple forms of cancer,^15^, ^16^, ^17^ in addition to being upregulated in HCC,^18^ and its overexpression has been reported to be predictive of overall survival (OS) and disease‐free survival in individuals with HCC.^19^ HULC‐mediated HBx upregulation can promote STAT3 coactivation, thereby stimulating miR‐539 promoter activation in the context of HBV‐related HCC.^20^ HULC can also serve as a diagnostic biomarker in HCC patients.^21^, ^22^, ^23^ Zhao et al. additionally determined that circulating HULC levels were increased in individuals suffering from cirrhosis associated with HBC.^24^

Autophagy is an essential regulator of cellular physiology and tumor development,^25^, ^26^, ^27^ and several autophagy‐associated lncRNAs have been shown to be of prognostic utility in various forms of cancer.^28^ HULC may play an important role in the onset of drug resistance in HCC,^29^ and HULC is expressed at higher levels in drug‐resistant gastric cancer cells in a manner correlated with decreased survival.^30^

Here, we analyzed HULC upregulation in human HCC and determined that this lncRNA can drive malignant progression in part via activating NF‐κB signaling in an autophagy‐dependent manner in these liver cancer cells. Through these analyses, we sought to clarify a novel mechanism whereby HULC governs liver cancer cell malignancy.

## 2 MATERIALS AND METHODS

### 2.1 Human tissue samples and data mining

Tumor tissues were collected from 50 HCC patients undergoing surgical tumor resection between January 2012 and May 2013 at the Affiliated Hospital of Qingdao University, China. Donors were an average of 55.66 years old (range: 21–87 years). All tumors were graded independently by two pathologists using the tumor, node, metastasis (TNM) staging system of the American Joint Committee (8th edition) as well as the Barcelona clinic liver cancer staging system. After ethical review, all subjects agreed to donate the diseased materials to the laboratory and provided corresponding written informed consent. After collection, tumor samples were snap‐frozen and stored at −80°C. Patients were followed for an average of 35.8 months after surgery (range: 1.2–66.8), and OS rates were measured. All study approaches were consistent with the standards set by the Declaration of Helsinki.

### 2.2 Data acquisition

To analyze differential HULC expression between normal liver tissues and liver cancer tissues, we generated a HULC differential plot using the TIMER website (https://cistrome.shinyapps.io/timer/).^31^ For pan‐cancer analyses of HULC, the TCGA analysis in the TIMER database was used. The UALCAN database (http://ualcan.path.uab.edu/) contains RNA‐seq and clinical data from 31 cancer types selected from the TCGA database.^32^

### 2.3 HCC cell culture, vector construction, and transfection

HepG2, Hep3B, and Huh7 HCC cells as well as LX‐2 control hepatic stellate cells were purchased from Procell (Wuhan, China). Cells were grown in RPMI‐1640 (Gibco, CA, USA) containing 10% fetal bovine serum (FBS) (Invitrogen, CA, USA) at 37°C in a humidified incubator containing 5% CO_2_. A HULC overexpression vector was constructed by amplifying this lncRNA and cloning into the HindIII and EcoRI sites of the pcDNA3.1(+) vector, yielding pcDNA3.1‐lncRNA HULC (short for OV‐HULC) as previously reported.^33^ The primers used in this experiment are shown in Table S1. Cells were transfected with pcDNA3.1(+) (Mock), pcDNA3.1‐lncRNA HULC (OV‐HULC), HULC‐specific or control siRNAs (siHULC and siMock; GenePharma, Shanghai, China) using Lipofectamine 3000 (Invitrogen, Carlsbad, CA, USA) based on the manufacturer's protocol.

### 2.4 RNA extraction, cDNA synthesis, and quantitative real‐time PCR (qPCR)

RNAiso (TaKaRa, Kyoto, Japan) was based on provided directions to extract RNA from samples of interest, after which a Reverse Transcription Kit (TaKaRa, Kyoto, Japan) was used to prepare cDNA from 1 μg of RNA per sample. All qPCR reactions were then conducted with a SYBR® Premix Ex Taq™ II kit (TaKaRa, Kyoto, Japan) according to the manufacturer's protocol with an FTC‐3000 instrument (Funglyn Biotech, Canada). Primers used for this study are shown in Table S1. GAPDH served as a normalization control. Relative gene expression was calculated using the 2^−ΔΔCt^ method.

### 2.5 Real‐time cell analysis

An xCELLigence Real‐Time Cell Analysis System (RTCA; ACEA Biosciences, CA, USA) in a humidified 37°C 5% CO_2_ incubator was used to monitor HCC cell migration. Briefly, the cells (5 × 10^4^/well) were added to the instrument chamber (E‐plate16, ACEA Biosciences), with appropriate treatments being applied. Cells were then monitored over a 20 h period, with electrical impedance being measured with gold microelectrodes to detect proliferation. This impedance was used to calculate the cell index value corresponding to cell proliferation, with this value being calculated every 10 min for the 20 h period with the RTCA software (ACEA Biosciences).

### 2.6 Colony formation assay

Hep3B and Huh7 cells were cultured and harvested using trypsin, diluted to 1000 cells/mL, and an average of 500 cells was added per well of a sixwell plate. After incubation in a tissue culture incubator for 2 weeks, colonies were then fixed using methanol (1 ml) for 10 min. The colonies were stained for 5 min with 0.1% crystal violet (Beyotime, China), washed under running water, counted, and analyzed.

### 2.7 Migration and invasion assays

Wound healing assays were performed to assess cellular migration. Briefly, 1 × 10^5^ cells were plated in 24‐well plates, and a pipette tip was used to generate a scratch in the monolayer surface. Cells were then rinsed with PBS, and wound sites were imaged at the indicated time points.

Transwell inserts were used to assess cellular migration (24‐well inserts, 8 μm pore size; Corning, NY, USA). At 48 h post‐transfection, cells were added to the upper chamber of these inserts (2 × 10^5^ cells/well) in RPMI‐1640 containing 2% FBS. The lower chamber was then filled with 500 μl of media containing 10% FBS. Cells were then incubated for 24 h, after which cells in the upper chamber were removed and those in the bottom chamber were fixed for 30 min in 4% paraformaldehyde after which DAPI (Sigma‐Aldrich, MO, USA) was used to stain the remaining cells. Migratory cells were then counted in five random fields of view using ImageJ (Media Cybernetics, Bethesda, MD, USA).

The protocol for cellular invasion assays was largely identical to that for migration assays, with the top chamber having first been coated using Matrigel (BD Biosciences, CA, USA), and with 1 × 10^5^ cells being introduced to the upper chamber. A fluorescent microscope (Celenas, Nikon, Tokyo, Japan) was used to quantify the results of this assay.

### 2.8 Western blotting

Proteins were extracted from the indicated cells using a Beyotime protein extraction kit, followed by the quantification of the protein levels in these samples using a bicinchoninic acid kit (Qiagen, CA, USA). A total of 30 μg of protein per sample was then separated via 10% SDS‐PAGE and transferred onto PVDF membranes (Millipore Corp., Bedford, MA, USA). These blots were then blocked with 5% non‐fat milk at 37°C for 2 h, after which they were probed overnight with antibodies from Cell Signaling Technology (Danvers, MA) specific for human LC3 (1:500, Cat. #12741), P62 (1:1000, Cat. #88588), Beclin1 (1:500, Cat. #3495), P65 (1:500, Cat. #3034), p‐P65 (Ser536) (1:500, Cat. #3033), IkBkB (1:1000, Cat. #8943), p‐IkBkB (Ser176/180) (1:500, Cat. #2694), and GAPDH (1:1000, Cat. #2118) at 4°C. Blots were then incubated with appropriate HRP‐conjugated secondary antibodies (Abcam, Cambridge, MA, USA) for 1 h at 37°C, after which protein levels were quantified via enhanced chemiluminescence (Pierce Biotechnology, Rockford, IL, USA) using Image Lab 2.0 (Bio‐Rad Laboratories, Hercules, CA, USA).

### 2.9 Electron microscopy

Following fixation for 4 h at 4°C using 2.5% glutaraldehyde (Solarbio, China), cells were rinsed in PBS and post‐fixed for 2 h using 1% OsO_4_ at 4°C. Cells were then rinsed and dehydrated with an ethanol gradient prior to embedding with Epon812 epoxy resin. Ultrathin (90 nm) sections were then collected on copper grids, dual‐stained with 1% uranyl acetate and 0.2% lead citrate, and imaged with a JEOL‐1200EX transmission electron microscope (Japan).

### 2.10 Immunofluorescence

The LV‐GFP‐RFP‐LC3 lentivirus (Hanbio, Shanghai, China) was used to transduce cells, which were subsequently transfected with OV‐HULC or siNC/siHULC constructs. Cells were then applied to the glass slides, fixed for 15 min with 4% paraformaldehyde (Boster), treated for 30 min with 5% bovine serum albumin (Sigma‐Aldrich, MO, USA), and stained for 7 min with (DAPI, Beyotime, China). Cells were imaged via confocal laser scanning microscopy (Leica Microsystems, Wetzlar, Germany). Numbers of GFP‐RFP‐LC3 punctae were detected in five independent fields of view.

### 2.11 Animal studies

The protocols of the Institutional Animal Care and Use Committee (IACUC) of the Affiliated Hospital of Qingdao University were used to guide the design of all animal studies, which received approval from the Affiliated Hospital of Qingdao University IACUC committee (AHQU‐MAL20190356). For these experiments, BALB/c athymic nu/nu mice (Vital River, 5 weeks old) received an injection in the left liver lobe of Huh7 cells (5 × 10^6^) stably expressing luciferase that had been transfected with siMock or siHULC constructs. Cells were injected in a 100 μl volume containing a 1:1 mixture of culture medium and growth factor‐reduced Matrigel. Orthotopic HCC xenograft tumor growth was measured every week after injection by injecting anesthetized mice with D‐Luciferin (100 μg/g), with a NightOWL II LB 983 System (Berthold, Bad Wildbad, Germany) then being used for murine imaging. The IndiGO2 software was used for luminescence calculations. At 4 weeks following tumor injection, nude mice were euthanized via carbon dioxide inhalation followed by cervical dislocation and the xenograft tumors were collected for histological and immunohistochemical staining.

### 2.12 Immunohistochemistry (IHC)

Standard protocols were used to deparaffinize and hydrate HCC tumor tissue sections (5 μm), after which a citrate buffer (pH 6) was used for antigen retrieval. The following primary antibodies were used for sample staining: Ki67 (1:50, Cat. #9449, Cell Signaling Technology, Danvers, MA), MET (1:50, Cat. #8198, Cell Signaling Technology, Danvers, MA), P65 (1:50, Cat. #8242, Cell Signaling Technology, Danvers, MA), and IkBkB (1:50, Cat. #8943, Cell Signaling Technology, Danvers, MA), with PicTure PV6000 and Elivision Plus (Zhongshan Chemical Co., Beijing, China) being used for staining and imaging.

### 2.13 Statistical analysis

All analyses were conducted using SPSS 19.0 (SPSS, IBM, Armonk, NY, USA) and GraphPad Prism 6 (GraphPad, CA, USA). Correlations between HULC expression and patient clinicopathological features were assessed using chi‐squared tests. Other data were compared using one‐way ANOVAs or Student's *t*‐tests, with *p* < 0.05 as the significance threshold.

## 3 RESULTS

### 3.1 HULC overexpression is correlated with a poor HCC patient prognosis

**FIGURE 1:**
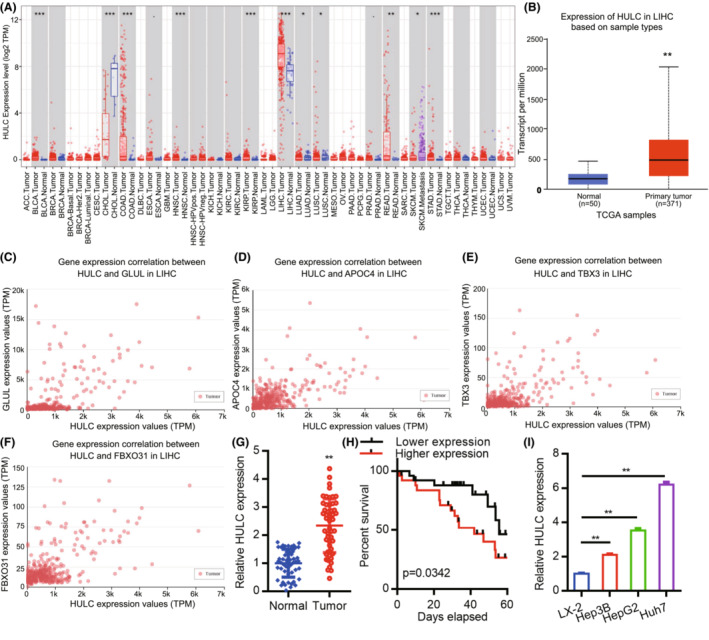
Analysis of HCC patient HULC expression levels. (A) Patterns of HULC expression across normal tissues and paired tumor samples, with individual dots corresponding to a specific sample. (B) HULC mRNA levels as measured in normal and HCC tumor tissues (**p < 0.01). (C–F) Genes identified as being positively correlated with HULC in HCC using the UALCAN tool. (G) HULC expression levels were assessed via qPCR in 50 HCC tumor and normal hepatic tissue samples. (H) Analysis of overall survival of patients with different HULC expression. (I) HULC expression levels in three HCC cell lines and one control hepatic stellate cell line (LX‐2) were assessed by qPCR, with GAPDH being used for normalization; *p < 0.05; **p < 0.01; ***p < 0.001.

We began by using the TIMER database to assess the expression of HULC across common cancer types (Figure 1A), revealing significant upregulation of this lncRNA in liver hepatocellular carcinoma (LIHC). A UALCAN analysis was then used to assess the expression of HULC in 50 normal tissue samples and 371 HCC patient tumor tissue samples from the TCGA database (Figure 1B). Further UALCAN analysis indicated that HULC expression levels were positively correlated with those of GLUL, APOC4, TBX3, and FBXO31 (Figure 1C–F). HULC levels were additionally assessed by qPCR in 50 HCC patient tumors and paired normal tissue samples, including 39 clinical stage I/II cases and 11 clinical stage III/IV cases (Table 1). This analysis revealed significant differences in HULC expression between these sample types (Figure 1G; *p* < 0.01). We then analyzed the relationship between HULC and the clinicopathological features of HCC. As shown in Table 2, strong associations were observed between HULC expression and liver cirrhosis (*p* = 0.018). However, the expression of HULC was not associated with age (*p* = 0.713), gender (*p* = 0.713), alcoholism (*p* = 0.758), α‐fetoprotein (AFP) level (*p* = 1.000), alanine aminotransferase (ALT) (*p* = 0.225), aspartate aminotransferase (AST) (*p* = 0.370), tumor number (*p* = 0.544), tumor size (*p* = 0.774), portal vein invasion (*p* = 0.269), or TNM stage (*p* = 0.733). Kaplan–Meier survival curves demonstrated that the overall survival of patients exhibiting low expression of HULC was significantly longer than that of patients with high levels of HULC expression (Figure 1H, *p* = 0.0342). Similarly, significant increases in HULC expression were observed in human HCC cell lines (Hep3B, HepG2, and Huh7) as compared to the control LX‐2 hepatic stellate cell line (Figure 1I). These results thus confirmed that HULC is significantly upregulated in HCC tumor tissues and cell lines.

**TABLE 1:** Clinicopathological characteristics of patient samples and expression of HULC in HCC

Characteristics	No. of case (%)
Age	
<50	9 (18.0)
≥50	41 (82.0)
Gender	
Male	41 (82.0)
Female	9 (18.0)
Alcoholism	
Yes	15 (30.0)
No	35 (70.0)
Liver cirrhosis	
Yes	32 (64.0)
No	18 (36.0)
AFP (ng/L)	
<200	36 (72.0)
≥200	14 (28.0)
ALT (U/L)	
<60	34 (68.0)
≥60	16 (32.0)
AST (U/L)	
<40	33 (66.0)
≥40	17 (34.0)
Tumor number	
Single	34 (68.0)
Multiple	16 (32.0)
Tumor size	
<5 cm	29 (58.0)
≥5 cm	21 (42.0)
Portal vein invasion	
Yes	9 (18.0)
No	41 (82.0)
TNM stage	
I+II stage	39 (78.0)
III+IV stage	11 (22.0)

Abbreviations: AFP, α‐fetoprotein; ALT, alanine aminotransferase; AST, aspartate aminotransferase; HCC, hepatocellular carcinoma; TNM, tumor, node, metastasis.

**TABLE 2:** Correlation between HULC expression and clinicopathologic characteristics of HCC patients

Characteristics HULC	HULC expression		
	Low or none, no. cases	High, no. cases	*p* value
Age			
<50	4	5	0.713
≥50	21	20	
Gender			
Male	21	20	0.713
Female	4	5	
Alcoholism			
Yes	8	7	0.758
No	17	18	
Liver cirrhosis			
Yes	20	12	**0.018**
No	5	13	
AFP (ng/L)			
<200	18	18	1.000
≥200	7	7	
ALT (U/L)			
<60	19	15	0.225
≥60	6	10	
AST (U/L)			
<40	18	15	0.370
≥40	7	10	
Tumor number			
Single	18	16	0.544
Multiple	7	9	
Tumor size			
<5 cm	14	15	0.774
≥5 cm	11	10	
Portal vein invasion			
Yes	3	6	0.269
No	22	19	
TNM stage			
I+II stage	19	20	0.733
III+IV stage	6	5	

*Note*: *p* values were calculated using chi‐square test. Bold numbers indicate significant differences (*p* < 0.05).

Abbreviations: AFP, α‐fetoprotein; ALT, alanine aminotransferase; AST, aspartate aminotransferase; HCC, hepatocellular carcinoma; TNM, tumor, node, metastasis.

### 3.2 HULC drives in vitro proliferation and metastatic activity in HCC cell lines

**FIGURE 2:**
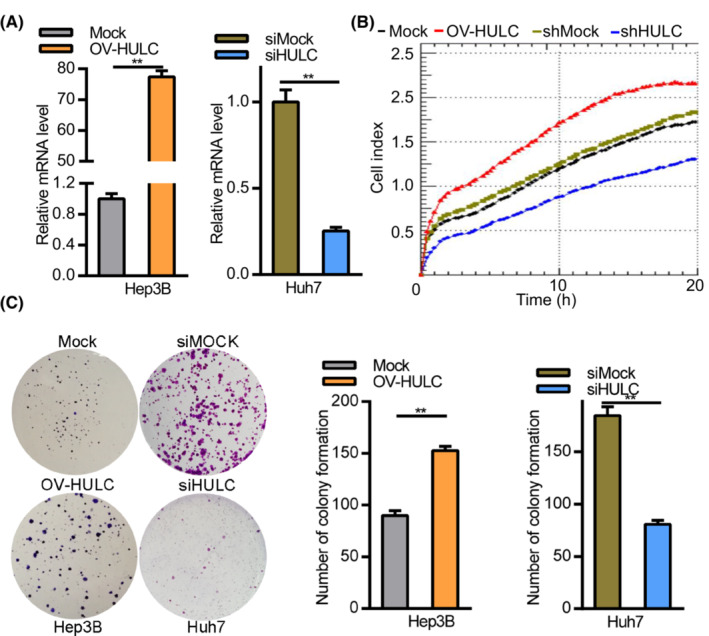
HULC expression is closely associated with HCC cell proliferation. (A) The knockdown and overexpression of HULC in Huh7 and Hep3B cells, respectively, were assessed by qPCR. *p* < 0.01; (B) Huh7 and Hep3B cell proliferation was assessed by RTCA assay following HULC overexpression or knockdown. (C) The effects of HULC expression on HCC cell proliferation were assessed via colony formation assay; *p* < 0.01.

Of the three tested HCC cell lines (Hep3B, HepG2, and Huh7) overexpressing HULC, Huh7 expressed the highest levels of HULC and also exhibited the most robust proliferative activity. Hep3B cells expressed the lowest levels of HULC in three tested HCC cell lines. To assess the functional importance of HULC among the HCC cell lines, we generated a HULC overexpression vector (OV‐HULC), and confirmed that treating Hep3B cells with this vector was sufficient to enhance HULC expression. Huh7 cells exhibited decreased HULC expression upon LV‐shHULC treatment (Figure 2A). An RTCA assay subsequently indicated that HULC overexpression resulted in enhanced HCC cell proliferation (Figure 2B). Consistent with these data, HULC overexpression was associated with increased Hep3B cell colony formation as compared to mock control cells (Figure 2C).

**FIGURE 3:**
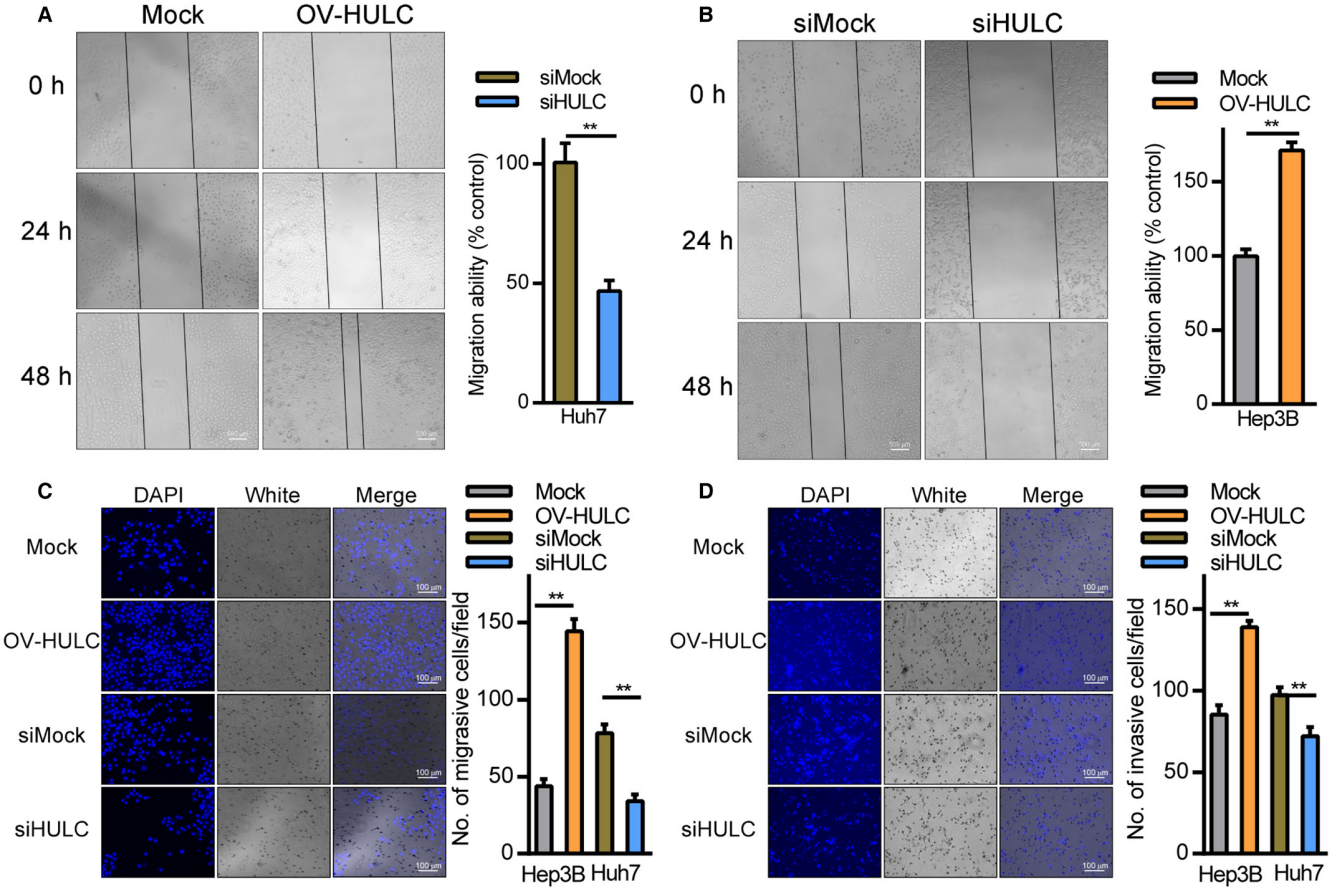
HULC impacts HCC cell migratory and invasive activity. (A, B) Wound healing assays were performed in HCC cells in which HULC was (A) overexpressed in Hep3B cells and (B) knocked down in Huh7 cells. Scale bar: 500 μm. (C, D) Transwell‐ (C) migration and (D) invasion assays were conducted using Hep3B and Huh7 cells in which HULC had been overexpressed and knocked down, respectively; scale bar: 100 μm; **p < 0.01.

The relationship between HULC expression and HCC cell migration was assessed through wound healing and Transwell assays. In wound healing assays, Hep3B cells overexpressing HULC exhibited more rapid wound closure relative to mock controls (Figure 3A), whereas HULC knockdown in Huh7 cells impaired wound closure (Figure 3B). Transwell assays further confirmed the ability of HULC overexpression to promote Hep3B cell migration and invasion, while HULC knockdown in Huh7 cells had the opposite effect (Figure 3C,D). These findings suggest that HULC can promote HCC cell metastasis and proliferation in vitro.

### 3.3 HULC influences autophagic activity in HCC cells

**FIGURE 4:**
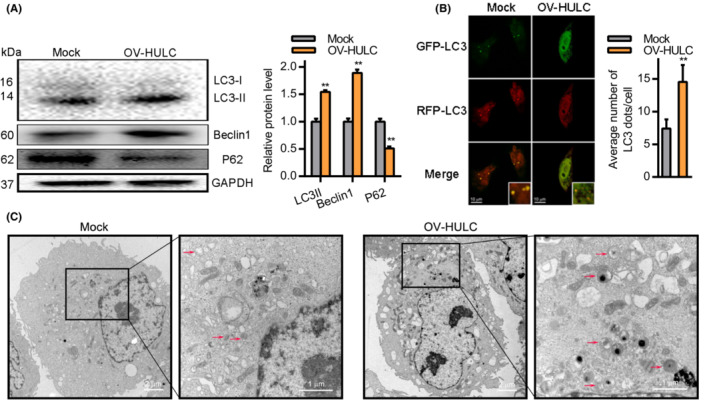
HULC overexpression impacts autophagic activity in HCC cells. (A) Following OV‐HULC transfection, the expression of LC3II, Beclin1, and P62 was measured in Hep3B cells by Western blotting, with GAPDH as a loading control. (B) Following OV‐HULC and Ad5‐GFP‐RFP‐LC3 transfection for 24 h, Hep3B cells were subjected to immunofluorescent staining. Scale bar, 10 μm. (C) Following OV‐HULC transfection for 24 h, Hep3B cells were assessed using electron microscopy. Scale bar, 2 μm (left) and 1 μm (right). Data are means ± SD from triplicate experiments; **p < 0.01.

Prior studies have indicated that HULC can modulate autophagic activity by regulating a variety of related pathways and proteins.^29^, ^34^ We thus tested the ability of HULC overexpression to drive autophagy within HCC cells. At 24 h post‐OV‐HULC transfection, LC3II and Beclin1 expression levels were increased whereas levels of P62 were significantly lower (Figure 4A). Consistent with these results, immunofluorescent staining indicated that there were significantly more LC3 punctae in Hep3B cells at 24 h post‐OV‐HULC transfection (Figure 4B), at which time significantly more autophagic vesicles were evident in these cells (Figure 4C). Together, these results indicate that overexpressing HULC can promote autophagosome formation in HCC cells.

### 3.4 Knockdown of HULC facilitates NF‐kB pathway activation in HCC cells

**FIGURE 5:**
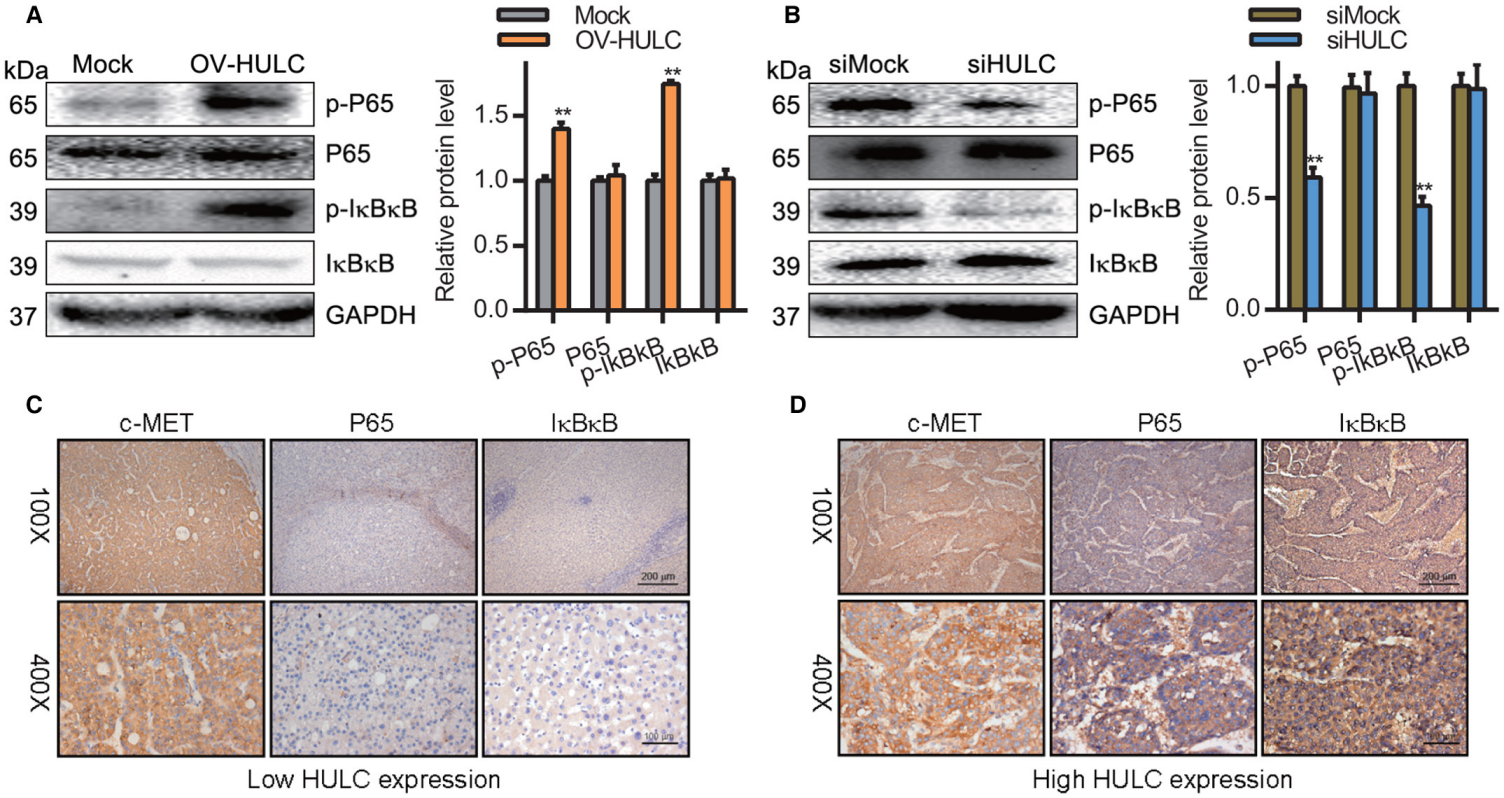
HULC downregulation inhibits NF‐kB pathway activation. (A, B) p‐p65 and p‐IκBκB levels were assessed by Western blotting following OV‐HULC transfection in (A) Hep3B cells and (B) Huh7 cells, with GAPDH as a normalization control. Quantification results are derived from three independent analyses. (C, D) Immunohistochemistry was used to measure c‐MET, P65, and IκBκB levels in HCC patients; **p < 0.01 versus Control.

The NF‐kB pathway plays a vital role in regulating inflammatory pathways as well as cellular proliferation, differentiation, autophagy, and related mechanisms.^35^ NF‐kB pathway activation in HCC can regulate tumor cell migration and proliferation.^36^ We evaluated the levels of p‐p65 and p‐IκBκB in HCC cells via Western blotting and found that HULC overexpression was associated with a significant increase in p‐p65 and p‐IκBκB levels, whereas the opposite was observed following HULC knockdown (Figure 5A,B **)**. To confirm these results, we assessed p65 and IκBκB levels in HCC patient tumor tissue samples through IHC staining, revealing both to be negatively correlated with the expression of HULC (Figure 5C,D). In light of these results, it is apparent that HULC can promote HCC cell metastasis in part by driving NF‐κB pathway activation.

### 3.5 HULC silencing impairs the in vivo growth of HCC tumors by disrupting NF‐kB pathway activation

**FIGURE 6:**
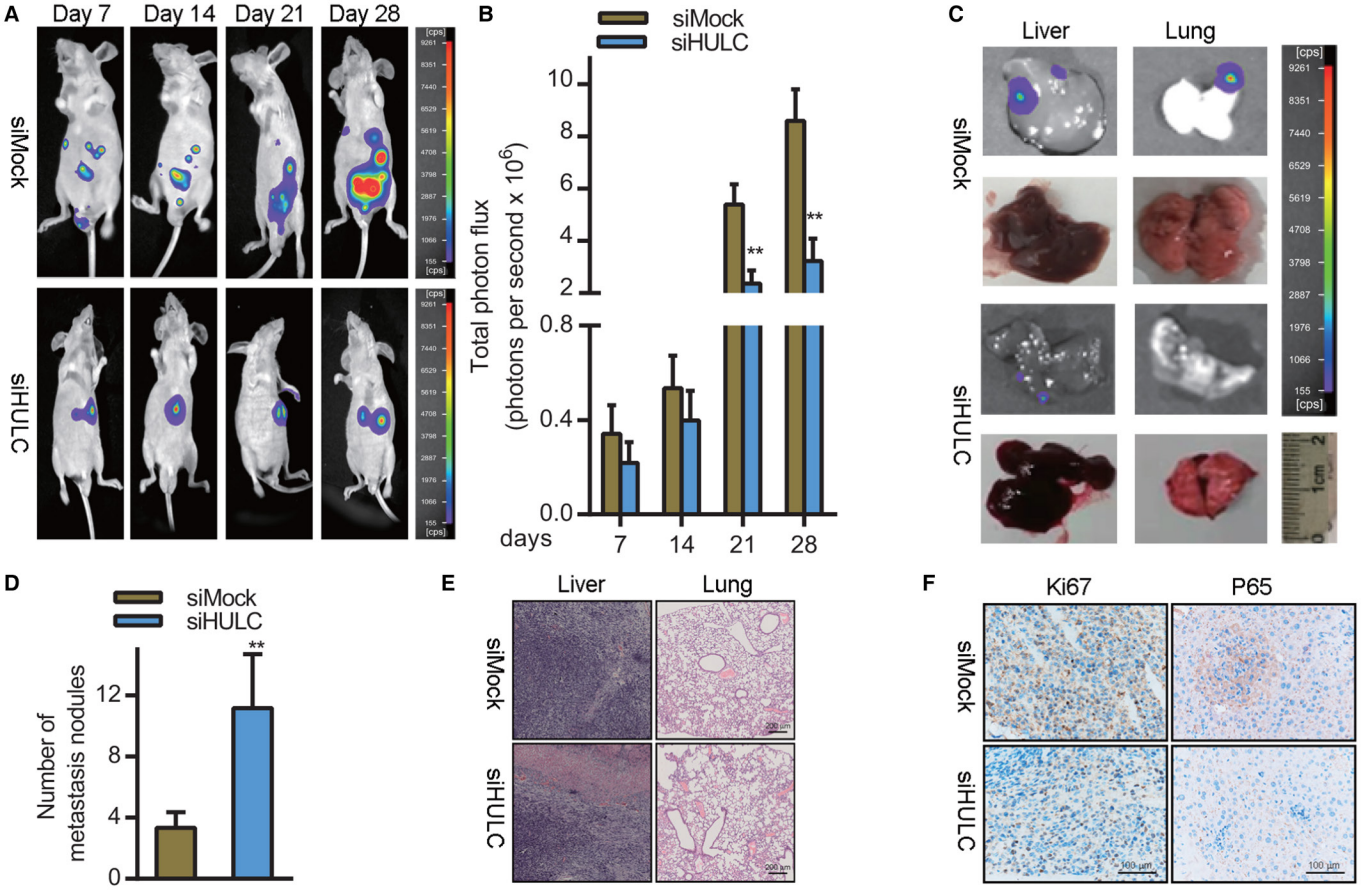
HULC knockdown impairs the growth of xenograft tumors by suppressing NF‐kB pathway activation. (A, B) Nude mice were injected with firefly luciferase‐labeled Huh7 cells transfected with mock (siMock) or HULC‐specific (siHULC) siRNA constructs in order to generate an orthotopic HCC xenograft model (n = 6/group). Tumor lung metastases in these mice were monitored on a weekly basis by injecting animals with luciferin and using a NightOWL LB 983 imaging system. (C) NightOWL LB 983 imaging‐based detection of pulmonary and hepatic metastases. (D) Representative pulmonary and hepatic lung nodule images. (E) Representative images of pulmonary and hepatic metastases following hematoxylin and eosin (H&E) staining (scale bar, 200 μm). (F) Immunohistochemistry‐based assessment of the effects of siHULC on Ki‐67 and P65 protein expression (scale bar, 100 μm).

Lastly, we explored the clinical relevance of HULC. To that end, we established an orthotopic HCC xenograft model by implanting nude mice with Huh7 cells transfected with siHULC or control constructs in the left lobe of the liver. HULC knockdown was associated with significant reductions in xenograft tumor growth relative to siMOCK‐transfected tumors (*p < 0.05) (Figure 6A,B). Liver and lung imaging revealed that tumor growth and lung metastasis were both impaired following HULC knockdown (Figure 6C). Consistently, H&E staining confirmed that there were significantly fewer pulmonary nodules in mice bearing siHULC‐transfected tumors relative to those bearing scramble control tumors, consistent with impaired lung metastasis (Figure 6D,E). Xenograft tumors in the siHULC group exhibited reduced immunohistochemical staining for the proliferation marker Ki‐67 (Figure 6F). Furthermore, expression of the NF‐kB pathway marker p65 was significantly downregulated in tumors from mice in the siHULC group (Figure 6F). Together, these data suggested that the knockdown of HULC can suppress in vivo HCC tumor growth at least in part by suppressing the activation of the NF‐kB pathway.

## 4 DISCUSSION

HULC is known to be an oncogenic lncRNA.^20^, ^29^, ^34^, ^37^ In our study, we explored the role of HULC in the context of HCC cell malignancy. Overall, we determined that HULC promotes enhanced HCC cell growth in vivo (Figure 6). Overexpressing HULC enhanced the expression of autophagy‐related factors including Beclin1 and LC3II. HULC also increased p‐P65 and p‐IkBkB levels, thus indicating that it can drive autophagy‐mediated activation of the NF‐kB signaling pathway in liver cancer cells.

At over 200 nucleotides in length, lncRNAs can play important roles in the inhibition or oncogenic development of several forms of cancer including gastric cancer.^38^, ^39^

HULC has repeatedly been shown to drive tumorigenesis.^37^, ^40^ We found this lncRNA to be linked to HCC tumor development through experiments revealing HULC overexpression in human liver cancer tissues. HULC is thus a robust oncogenic lncRNA.

**FIGURE 7:**
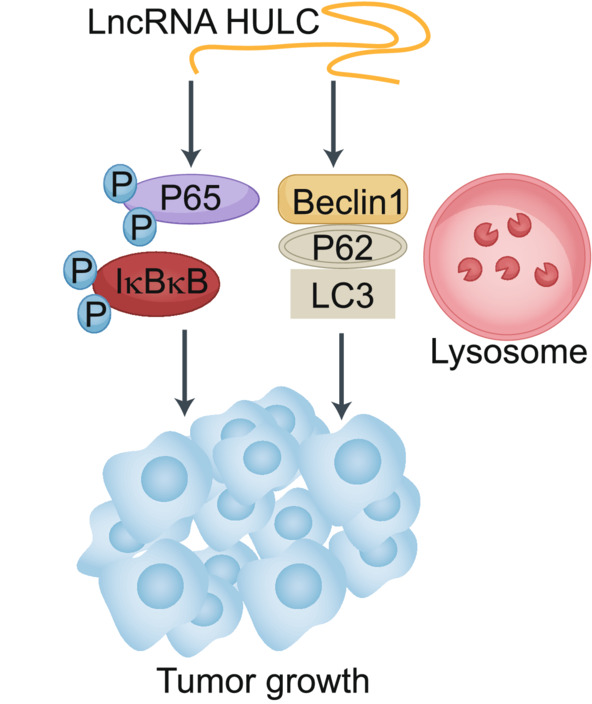
Proposed molecular model of the mechanisms governing HULC‐mediated promotion of HCC growth through the NF‐kB pathway and autophagy.

HULC can drive the proliferative, migratory, and invasive activity of cells in vitro. Consistent with such activities, we observed enhanced proliferation, migration, and invasion together with reduced apoptotic cell death in HCC cells upon HULC overexpression, whereas HULC knockdown had the opposite effect. HULC may thus function as an oncogenic lncRNA in this cancer type. We further determined that HULC is capable of promoting autophagic activity in HCC cells as evidenced by increased LC3 and Beclin1 expression together with P62 downregulation upon HULC overexpression. Immunofluorescent staining and electron microscopy further confirmed the ability of this lncRNA to drive autophagosome formation, indicating that HULC may drive oncogenesis via promoting autophagy (Figure 7).

The NF‐κB signaling pathway is closely linked to malignancy in HCC and other cancers.^41^, ^42^ Previous research has found that there is regulatory cross‐talk between autophagy and NF‐κB signaling pathways.^43^, ^44^ In line with such observations, we detected significantly elevated p‐p65 and p‐IkBkB levels in cells overexpressing HULC (Figure 7).

The present study demonstrated that HULC may play a role in HCC oncogenesis by promoting autophagy and thereby driving NF‐kB pathway signaling. We speculate that HULC may thus function as a valuable diagnostic biomarker and viable therapeutic target in this cancer type. However, more studies are essential to understand the potential clinical relevance of HULC in the inhibition of HCC and its role in oncogenesis.

There are two potential limitations to this study. First, all clinical analyses were retrospective in nature. Future prospective analysis of HCC tissue samples should be conducted to firmly establish the clinical importance of HULC. Secondly, while we assessed the role of HULC in HCC regulation, more in‐depth analysis is necessary to substantiate the mechanistic basis for these regulatory relationships.

## AUTHOR CONTRIBUTIONS

Shihai Liu, Lakshmi Huttad, and Jing Qiu: Study design, data analysis, data interpretation, and writing, and review of the article. Guifang He, Weitai He, and Duo Cai: Experiment excecution, data analysis, and review of the article. Hao Chen and Changchang Liu: Study design, data analysis, and review of the article. Lakshmi Huttad and Hao Chen: Study design, data collection, data analysis, data interpretation, and writing and review of the article.

## FUNDING INFORMATION

This work was supported by the Natural Science Basic Research Program Shaanxi Province (2020JM‐008), Natural Science Basic Research Program Shandong Province (ZR2021MH022), Natural Science Foundation of Zhejiang Province of China (LGF19H160014), Source Innovation Foundation of Qingdao (Grant No. 18‐2‐2‐79‐jch) and “Clinical Medicine + X” of Qingdao University (Grant No. CMX201729).

## CONFLICT OF INTEREST

We have no conflicts of interest to declare.

## ETHICS STATEMENT

This study was conducted according to the principles expressed in the Declaration of Helsinki. This study was approved by the Ethics Committee of the Affiliated Hospital of Qingdao University (2012‐ECAHQU‐006). All tissues were obtained with informed consent. The protocols of the Institutional Animal Care and Use Committee (IACUC) of the Affiliated Hospital of Qingdao University guidelines was used to guide the design of all animal studies, which received approval from the Affiliated Hospital of Qingdao University IACUC committee (AHQU‐MAL20190356).

## INFORMED CONSENT

This study was approved by the Ethics Committee of the Affiliated Hospital of Qingdao University (2012‐ECAHQU‐006). All tissues were obtained with informed consent.

## Supporting information


Table S1

## Data Availability

All data generated or analyzed during this study are included in this published article.
