# Supplementary material for: Long noncoding RNA HULC regulates the NF‐κB pathway and represents a promising prognostic biomarker in liver cancer
Source: Cancer Med. 2022 Oct 10;12(4):5124–36. doi: 10.1002/cam4.5263 (PMC9972175; doi:10.1002/cam4.5263)
Supplement: Supplementary file 1 — Table S1 [file CAM4-12-5124-s001.pdf]

Table 1. Clinicopathological characteristics of patient samples and expression of HULC in HCC

**Characteristics**	**No. of case (%)**
**Age**	
<50	9 (18.0)
≥50	41 (82.0)
**Gender**	
Male	41 (82.0)
Female	9 (18.0)
**Alcoholism**	
Yes	15 (30.0)
No	35 (70.0)
**Liver cirrhosis**	
Yes	32 (64.0)
No	18 (36.0)
**AFP (ng/L)**	
<200	36 (72.0)
≥200	14 (28.0)
**ALT (U/L)**	
<60	34 (68.0)
≥60	16 (32.0)
**AST (U/L)**	
<40	33 (66.0)
≥40	17 (34.0)
**Tumor number**	
Single	34 (68.0)
Multiple	16 (32.0)
**Tumor size**	
<5cm	29 (58.0)
≥5cm	21 (42.0)
**Portal vein invasion**	
Yes	9 (18.0)
No	41 (82.0)
**TNM stage**	
I+II stage	39 (78.0)
III+IV stage	11 (22.0)

HCC hepatocellular carcinoma, AFP α-fetoprotein, ALT alanine aminotransferase, AST aspartate aminotransferase, TNM tumor, node, metastasis

Table 2. Correlation between HULC expression and clinicopathologic characteristics of HCC patients

Characteristics HULC	HULC expression		
	Low or none, no. cases	High, no. cases	p value
**Age**			
<50	4	5	0.713
≥50	21	20	
**Gender**			
Male	21	20	0.713
Female	4	5	
**Alcoholism**			
Yes	8	7	0.758
No	17	18	
**Liver cirrhosis**			
Yes	20	12	**0.018**
No	5	13	
**AFP (ng/L)**			
<200	18	18	1.000
≥200	7	7	
**ALT (U/L)**			
<60	19	15	0.225
≥60	6	10	
**AST (U/L)**			
<40	18	15	0.370
≥40	7	10	
**Tumor number**			
Single	18	16	0.544
Multiple	7	9	
**Tumor size**			
<5cm	14	15	0.774
≥5cm	11	10	
**Portal vein invasion**			
Yes	3	6	0.269
No	22	19	
**TNM stage**			
I+II stage	19	20	0.733
III+IV stage	6	5	

P values were calculated using chi-square test. Bold numbers indicate significant differences ($P < 0.05$). HCC hepatocellular carcinoma, AFP α-fetoprotein, ALT alanine aminotransferase, AST aspartate aminotransferase, TNM tumor, node, metastasis

Supplementary table 1. Primer sequences for qPCR, siRNA

Genes	Primer direction	Primer sequence (5′ -3′)
HULC	qPCR primer F	AACCTCCAGAACTGTGAT
	qPCR primer R	CATAATTCAGGGAGAAAG
	pcDNA3.1-HULC primer F	CCCAAGCTTATGGGGGTGGAACTCATGATGGAAT
	pcDNA3.1-HULC primer R	GGAATTCAAGAATGGACATCATTTTATTTCA
	siRNA F	GGAAGAAACUCUGAAGUAAdtdt
	siRNA R	UUACUUCAGAGUUUCUUCCdtdt
GAPDH	qPCR primer F	ACTGCCACCCAGAAGACT
	qPCR primer R	GCTCAGTGTAGCCCAGGAT
